# A Cyclophilin OsCYP20–2 Interacts with OsSYF2 to Regulate Grain Length by Pre-mRNA Splicing

**DOI:** 10.1186/s12284-020-00425-0

**Published:** 2020-09-10

**Authors:** Qiang Ge, Yongyan Tang, Wei Luo, Jingyu Zhang, Kang Chong, Yunyuan Xu

**Affiliations:** 1 Key Laboratory of Plant Molecular Physiology, Institute of Botany, Chinese Academy of Sciences, Beijing, 100093 China; 2 University of Chinese Academy of Sciences, Beijing, 100049 China; 3 Present Address: College of Agronomy, Henan Agricultural University, Zhengzhou, 450046 China; 4 Innovation Academy for Seed Design, CAS, Beijing, 100101 China

**Keywords:** OsCYP20–2, Grain length, OsSYF2, Spliceosome, Alternative splicing

## Abstract

**Background:**

Grain size is one of the key agronomic traits that impact grain yield. Several regulatory pathways had been reported to participate in grain size determination via cell expansion or proliferation in rice. However, little is known about cyclophilin and spliceosome participation in grain shape regulation.

**Results:**

Here, we identified OsCYP20–2, a cyclophilin that influences spliceosome assembly to determine grain length. *oscyp20–2 t1*, a knock out mutant of *OsCYP20–2* caused by T-DNA insertion, produced shorter grains with deficient cell elongation. Through yeast two-hybrid screening and pull-down assays, OsSYF2, a pre-mRNA splicing factor, was identified as an interacting protein of OsCYP20–2. The phenotypes of transgenic lines indicated that *OsSYF2* positively regulates grain length via its influence on cell expansion. Transcriptomic analysis showed that *OsSYF2* controls the expression and pre-mRNA alternative splicing of genes involved in sugar metabolism. In addition, these two genes have similar effects on panicle architecture.

**Conclusions:**

Taken together, OsSYF2, an interacting protein of OsCYP20–2, controls grain length and panicle architecture by regulating the alternative splicing of pre-mRNA involved in cell elongation and sugar metabolism.

## Background

Vital agronomic traits for yield improvement in rice include inflorescence architecture and grain size. Genetic and molecular analyses have identified numerous quantitative trait loci (QTLs) and genes involved in multiple signaling pathways that regulate grain size. For example, G-protein signaling positively regulates grain length through the concerted actions of RGA1, RGB1, GS3, and DEP1 (Ashikari et al. 1999; Fan et al. 2006; Huang et al. 2009; Mao et al. 2010; Xu et al. 2019). The ubiquitin mediated protein degradation pathway (Chen et al. 2013; Huang et al. 2017; Song et al. 2007), the mitogen-activated protein kinase signaling pathway (Wang et al. 2019), and several hormone signaling pathways (Shirley et al. 2019; Xiao et al. 2019) also affect grain size.

The protein conformational change impacts protein folding and assembling to regulate its cellular functions (Zhang et al. 2019). Proline residues can form *cis* and *trans* peptide bonds. The prolyl *cis*-*trans* isomerization functions as a molecular switch to regulate protein conformation. This prolyl *cis*-*trans* conversion can be catalyzed by peptidyl prolyl *cis*-*trans* isomerase (PPIase). PPIases can be divided into four families: protein Ser/Thr phosphatase 2A (PP2A) activator, FK506-binding proteins (FKBPs), parvulins, and cyclophilins (CYPs) (Lu et al. 2007). CYPs are evolutionarily and structurally conserved proteins, and have been identified in both prokaryotes and eukaryotes. Cyclophilins are found in all cellular compartments and are involved in multiple processes. In plants, CYPs can regulate organogenesis (Fulgosi et al. 1998), hormone signaling (such as GA, and BR) (Li et al. 2010; Zhang et al. 2013), as well as defense responses (Berardini et al. 2001; Li et al. 2014; Trupkin et al. 2012). In *Arabidopsis*, the PPIase domain interacted with snRNP-specific proteins to regulate pre-mRNA splicing (Lorkovic et al. 2004). In rice, overexpression of the Golgi-resident OsCYP21–4 can increase yield by promoting the accumulation of mannosidic glycoproteins (Park et al. 2017). OsCYP20–2 could integrate chilling tolerance and cell elongation by its dual-localization. Under low temperature, OsCYP20–2 functions with OsFSD2 to scavenge reactive oxygen species (ROS) in chloroplasts, and the nuclear-localized variant OsNuCYP20–2 targets SLENDER RICE1 to regulate GA signaling (Ge et al. 2020). However, it remains unknown if CYPs regulate grain size by other mechanisms.

Alternative splicing is crucial for plant growth and stress response. The removal of introns from pre-mRNA is subtly modulated by a variety of splicing factors. Several nuclear-localized CYPs function in spliceosome assembly in humans (Adams et al. 2015; Galat 1993; Wang and Heitman 2005). Cyclophilin USA-CyP formed independent complexes with the splicing factor hPrp18 and hPrp4 to participate in pre-mRNA splicing (Horowitz et al. 2002). SYF2/NTC31/p29, is a critical splicing factor in human and yeast. Mutation of *SYF2* results in cell cycle arrest by activation of the spindle checkpoint in *Saccharomyces cerevisiae* (Dahan and Kupiec 2002). In humans, overexpression of *SYF2* promotes cell proliferation in breast cancer (Shi et al. 2017). However, the function of *SYF2* orthologs in plants is completely unknown.

Here, we demonstrated that OsCYP20–2 can interact with splicing factor OsSYF2 to impact grain length. Both the *oscyp20–2 t1* mutant and *OsSYF2* RNA interference (RNAi) lines exhibited shorter grain length than wild type. RNA-sequencing analysis demonstrated that *OsSYF2* was involved in pre-mRNA alternative splicing and transcriptional regulation of some sugar metabolism pathway genes. Thus, our findings revealed that OsCYP20–2 and OsSYF2 interact to determine grain size in rice.

## Results

### OsCYP20–2 Regulates Grain Size and Inflorescence Architecture

**Fig. 1:**
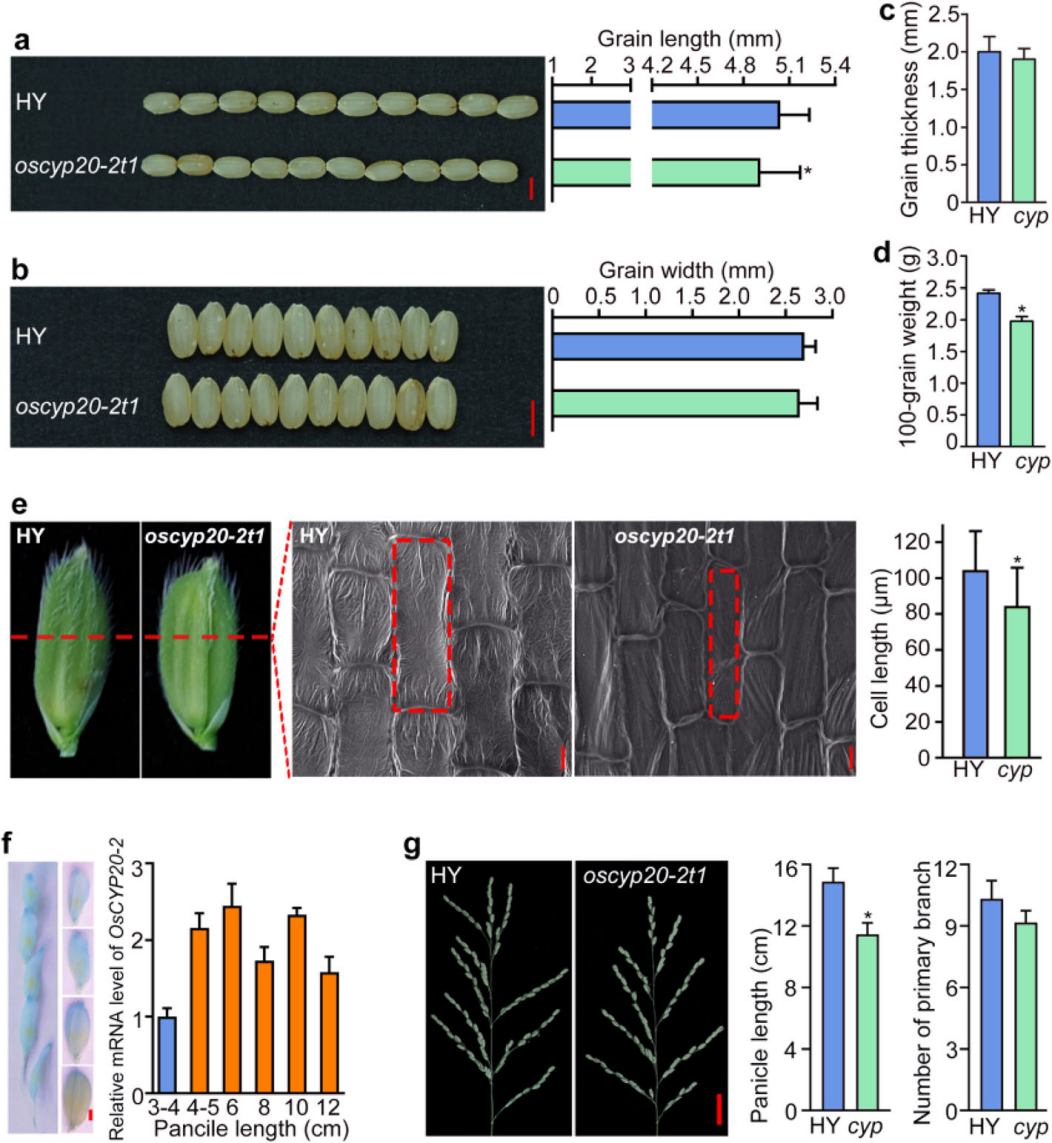
Phenotypes of knock-out mutant *oscyp20–2 t1*. **a** Comparison of brown grain length between wild type Hwayoung (HY) and *oscyp20–2 t1* (*cyp*)*.* At least 200 seeds were counted. Bar = 4 mm. *P < 0.05, Student’s *t* test. **b** Comparison of brown grain width between wild type HY and mutant *cyp.* Bar, 4 mm. Average brown grain width of HY and *oscyp20–2 t1*. At least 200 seeds were counted. **c** and **d** Statistics of brown grain thickness and 100-grain weight data of HY and *oscyp20–2 t1*, respectively. Error bar means SD. At least 200 seeds were measured. SD means six biological replicates in 100-grain weight statistics data. *P < 0.05, Student’s *t* test. **e** Cell morphology and average cell length of inner epidermal cells of lemmas in HY and *oscyp20–2 t1*. Scale bar, 20 μm. Error bar indicate SD for at least 30 cells. *P < 0.05, Student’s *t* test. **f** GUS staining of glume tissues of *pOsCYP20–2:GUS* transgenic plants at various stages of panicle from P2 to P6 stage (Jain et al. 2007) and qRT-PCR results of *OsCYP20–2* expressing in different length of panicle. The panicles with indicated lengths were sampled for the analysis. **g** Panicle morphology of HY and *oscyp20–2 t1* (*cyp*). The length of inflorescence and number of branches were compared between HY and *oscyp20–2 t1.* Scale bar, 2 cm. Error bar was calculated for at least 10 plants. *P < 0.05, Student’s *t* test

We previously reported that CYP20–2 regulated plant height and chilling tolerance in rice (Ge et al. 2020). Unexpectedly, we found that the mutation of *OsCYP20–2* also reduced grain length, while brown grain width and thickness were unchanged (Fig. 1a-c). Notably, the 100-grain weight of the knock-out mutant *oscyp20–2 t1* was substantially decreased compared to that of wild type Hwayoung (HY) (Fig. 1d). Moreover, the spikelet hull and the lemma epidermal cells of *oscyp20–2 t1* were shorter than that of HY (Fig. 1e). These observations suggested that OsCYP20–2 determines grain length via regulation of cell length.

Additionally, GUS staining of *OsCYP20–2* promoter-driven *GUS* transgenic plants showed that *OsCYP20–2* is highly expressed in the various stage of panicle from P2 to P6 (Jain et al. 2007) (Fig. 1f). Consistently, panicle length and branch number were decreased in *oscyp20–2 t1* compared to HY (Fig. 1g).

### OsCYP20–2 Physically Interacts with OsSYF2

**Fig. 2:**
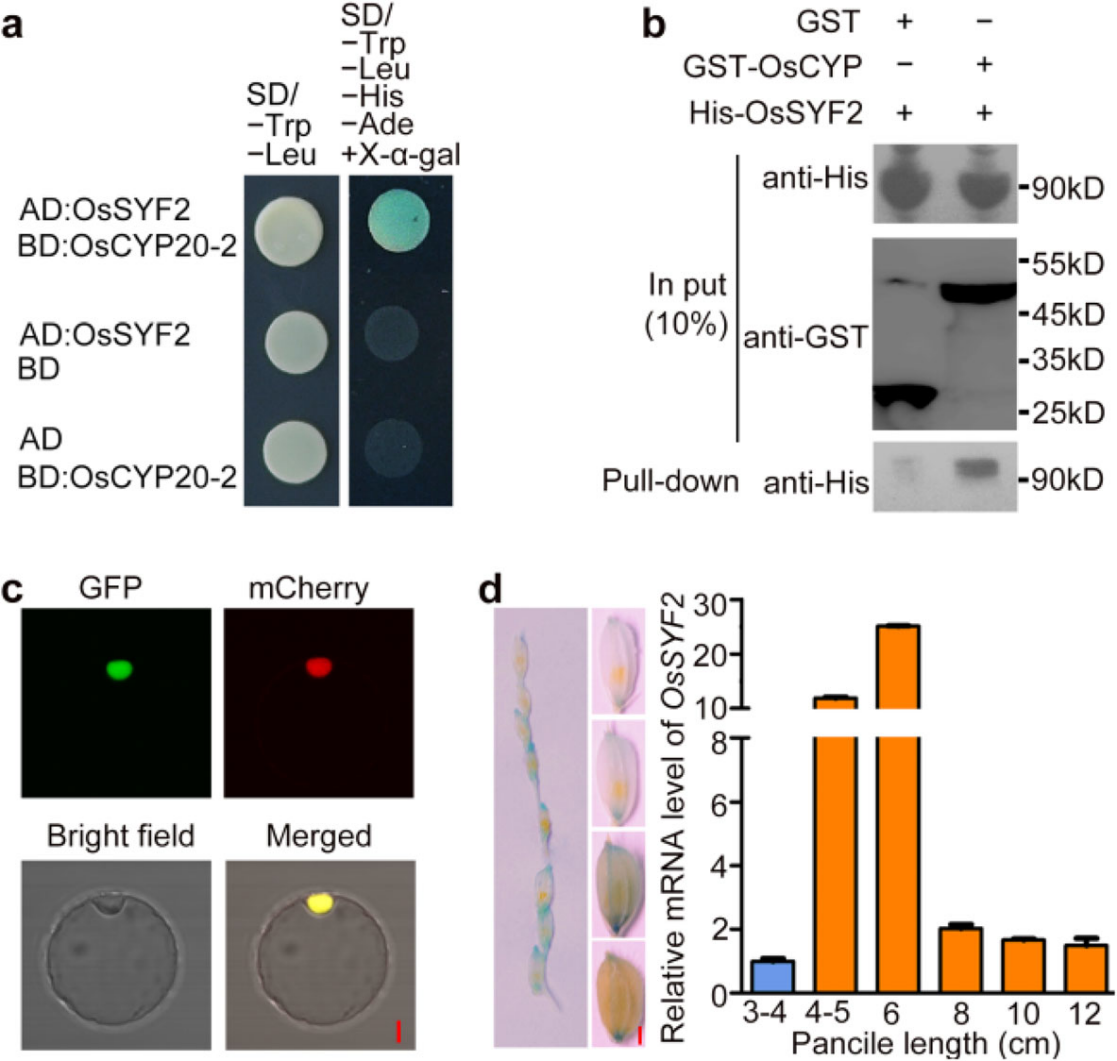
The interaction between OsCYP20–2 and OsSYF2. **a** OsCYP20–2 interacted with OsSYF2 in yeast. The clones were cultured in SD/-Trp-Leu-His-Ade medium containing 2 μg/mL X-α-gal. **b** GST pull-down assay identified the interaction of CYP20–2 with OsSYF2 in vitro. **c** Subcellular localization of *OsSYF2*. OsSYF2-GFP was co-expressed with H2B-mCherry in rice protoplasts. Bar = 50 μm. **d** Expression pattern of *OsSYF2*. The GUS staining of glume of *OsSYF2::GUS* transgenic plants (left) various stages of panicle from P2 to P6 stage (Jain et al. 2007) and transcript levels of *OsSYF2* at different length of panicles. The panicles with indicated lengths were sampled for the analysis

To search for potential interacting proteins of OsCYP20–2, the rice yeast two-hybrid cDNA library was screened using OsCYP20–2-BD as bait. An accessory spliceosome protein named Synthetic lethal with cdc forty 2 (OsSYF2) was identified. SYF2 was reported to participate in pre-mRNA splicing and gene expression regulation in yeast (Vincent et al. 2003). The interaction of OsCYP20–2 and OsSYF2 was further confirmed by yeast two-hybrid and pull-down assays (Fig. 2a, b). Furthermore, *OsSYF2* shared a similar expression pattern with *OsCYP20–2* (Figs. 1f and 2d). Fluorescent microscopy was used to show that OsSYF2-GFP fluorescence overlapped with the nuclear marker H2B-mCherry in rice protoplasts (Fig. 2c). These results indicated that OsSYF2 is nuclear-localized and might interact with OsCYP20–2 to regulate seed development.

### *OsSYF2* Positively Regulates Grain Length

**Fig. 3:**
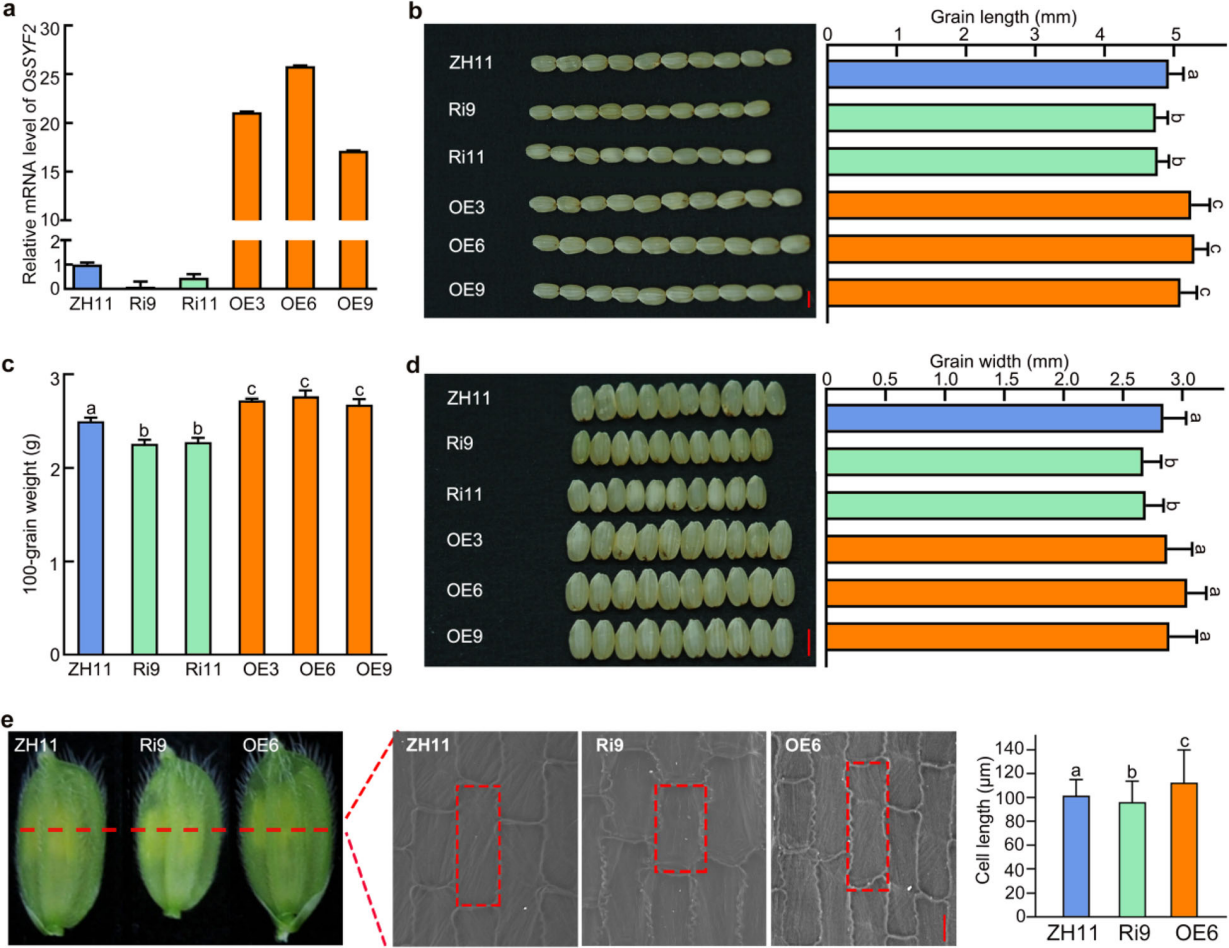
Effects of *OsSYF2* on grain size. **a** The expression levels of *OsSYF2* in Zhonghua 11 (ZH11), OsSYF2-overexpression (OE), and OsSYF2-RNAi (Ri) lines. **b** Grain length of ZH11 and *OsSYF2* transgenic lines*.* Bar = 4 mm. Brown grain length of ZH11 and *OsSYF2* transgenic plants. At least 200 seeds were counted per genotype. Letters indicate significant differences determined by a Least Significant Difference (LSD) test (*P* < 0.05). **c** Comparisons of 100-grain weight between ZH11 and *OsSYF2* transgenic plants. Error bar means SD. SD derived from six biological replicates in 100-grain weight measurements. Letters indicate significant differences determined by a LSD test (*P* < 0.05). **d** Grain width of wild type Zhonghua11 (ZH11) and *OsSYF2* transgenic plants*.* Bar = 4 mm. Brown grain width of ZH11 and *OsSYF2* transgenic material. At least 200 seeds were calculated. Letters indicate significant differences determined by a LSD test (*P* < 0.05). **e** Cell morphology and average cell length of inner epidermal cells of lemmas. Bar = 20 μm. Error bars indicate SD for at least 30 cells. Letters indicate significant differences determined by a LSD test (*P* < 0.05)

To confirm whether *OsSYF2* determines grain size or shape, the full-length CDS of *OsSYF2* under the control of maize *Ubiquitin 1* promoter was transformed into the *japonica* variety Zhonghua 11 (ZH11). Meanwhile, Os*SYF2* RNA interference lines (SYF2-Ri) were obtained by transforming a construct containing a 410-bp fragment targeting the second and third exons of *OsSYF2* (Fig. 3a) into ZH11. The *SYF2*-overexpression lines (SYF2-OE) displayed increased brown grain length and 100-grain weight compared to ZH11 (Fig. 3b, d). The RNAi lines Ri9 and Ri11 possessed similar grain phenotypes as *oscyp20–2 t1*, with decreased brown grain length, grain width, and 100-grain weight (Fig. 3b-d).

Subsequently, the cell size of epidermal cells in the outer glumes of OE6 and Ri9 lines were observed using scanning electron microscopy. Our results revealed that the cell length of OE6 was longer than that of ZH11, while Ri9 showed shorter cells compared to ZH11 (Fig. 3e). These results suggested that *OsSYF2* positively regulates grain size by increasing cell length.

### Knock-Down of *OsSYF2* Causes Similar Panicle Phenotypes as *oscyp20–2 t*

**Fig. 4:**
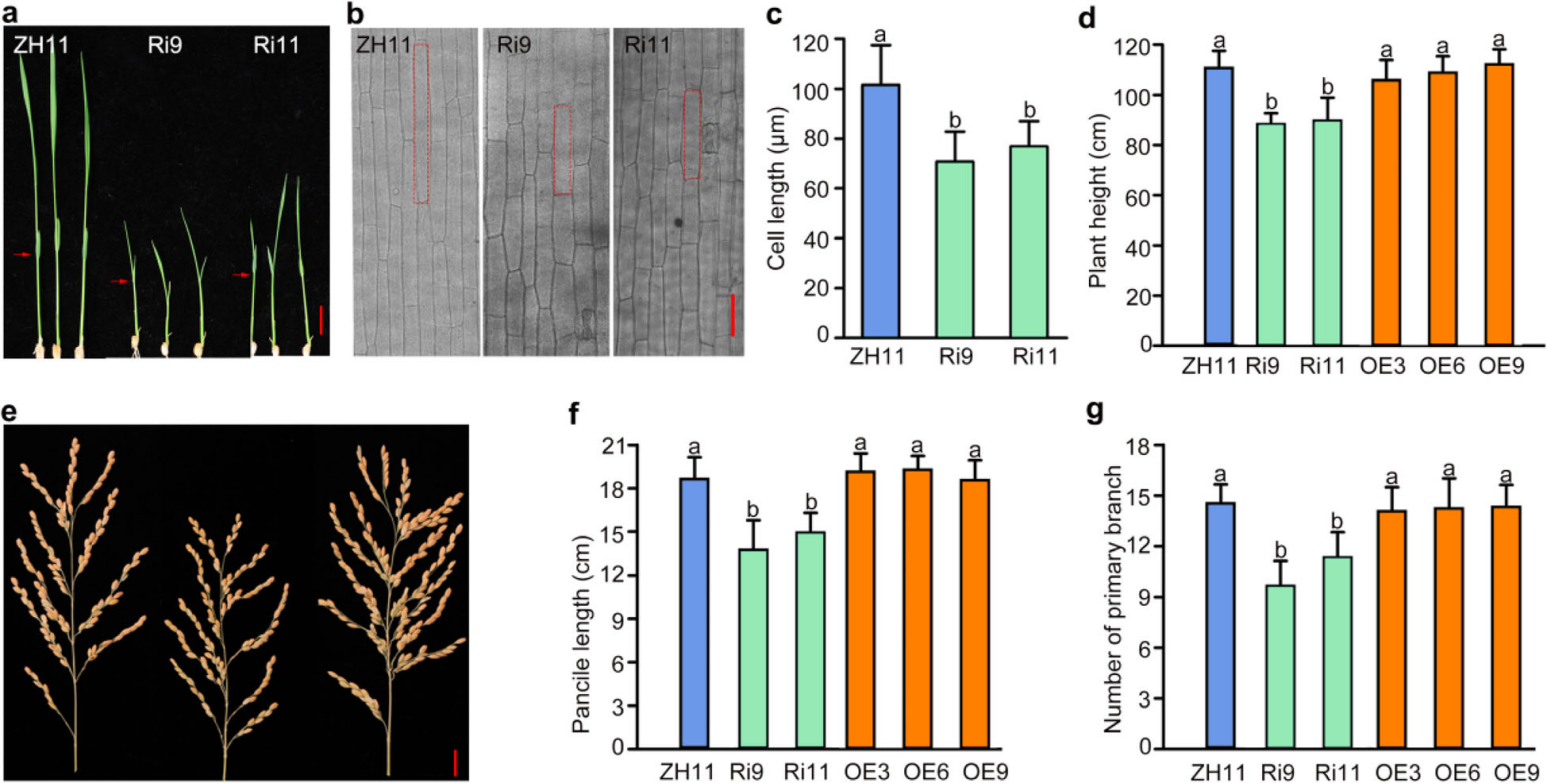
Agronomic traits of *OsSYF2* transgenic lines. **a** The phenotype of ZH11, Ri9 and Ri11 7 d seedings. Bar = 1 cm. **b** and **c** Morphology and statistical epidermal cell length of the second leaf sheath at three-leaf stage seedlings. Bar = 40 μm. Data are given as mean ± SD (*n* = 30). Letters indicate significant differences determined by a LSD test (*P* < 0.05). Letters showed the significant differences, which was ranked by Least Significant Difference (LSD) test (*P* < 0.05). **d** The plant height of ZH11 and *OsSYF2* transgenic plants measured at maturity. Error bar means SD, at least 7 plants were calculated. Letters indicate significant differences determined by a LSD test (*P* < 0.05). **e**-**g** The panicle morphology, inflorescence length, and the number of primary branches of ZH11 and *OsSYF2* transgenic plants at maturity, respectively. Error bar means SD. At least 10 plants were calculated. Bar = 1 cm. Letters indicate significant differences determined by a LSD test (*P* < 0.05)

Besides grain size, plant height and rachis length were also investigated in the transgenic lines. In *OsSYF2*-RNAi lines, plant height showed shorter than wild type ZH11 when seedlings were at three leaf stage (Fig. 4a). Cell size and number are the major factors that contribute to plant organ size. To explore the mechanism of *OsSYF2* regulation of plant height, we investigated the cell size of the second leaf sheath of wild type and RNAi lines at three leaf stage (Tang et al. 2018). The statistical results showed that the cell lengths in Ri9 and Ri11 were shorter than those of ZH11 (Fig. 4b, c).

In *OsSYF2*-RNAi lines, plant height, panicle length, and branch number were all decreased compared to ZH11 at mature stage. However, no significant changes were observed in *OsSYF2*-overexpression lines (Fig. 4d-g). Overall, down-regulating the expression of *OsSYF2* resulted in decreased plant height and panicle length.

### *OsSYF2* Associates with Sugar Metabolism

**Fig. 5:**
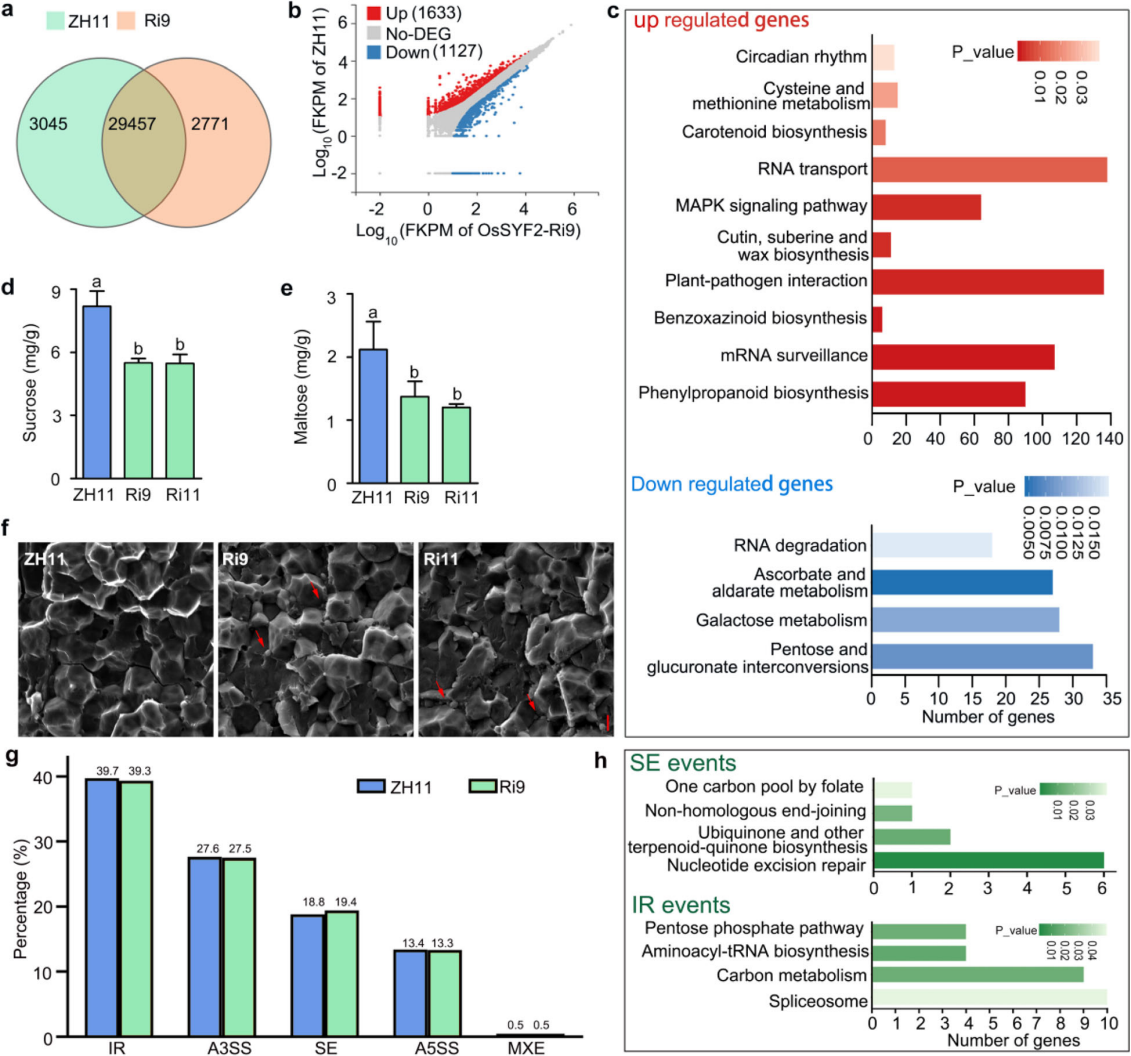
Analysis of RNA-seq between ZH11 and SYF2-RNAi9. **a** Overview of differentially-expressed genes between ZH11 and OsSYF2-RNAi9 transcriptomes. **b** Scatter-plot showing the differentially expressed genes between ZH11 and OsSYF2-RNAi9 transcriptomes. **c** Kyoto Encyclopedia of Genes and Genomes (KEGG) pathway enrichment analysis of genes expressed in ZH11 and OsSYF2-RNAi9 6 cm panicles. KEGG pathway analyses were applied to genes up-regulated or down-regulated between ZH11 and OsSYF2-RNAi9. Significant enrichment was considered with *P* value < 0.05. **d** and **e** Sucrose (**d**) and maltose (**e**) content in the mature endosperm of ZH11, Ri9, and Ri11. Data means SD (*n* = 3). Letters indicate significant differences determined by a LSD test (*P* < 0.05). **f** Starch morphology of ZH11 and *OsSYF2* transgenic plants detected by scanning electron microscopy. **g** The percentage of alternative splicing events between ZH11 and SYF2-RNAi9 revealed by RNA-Seq analysis. Alternative 5′ splice site (A5SS), alternative 3′ splice site (A3SS), mutually exclusive exon (MXE), intron retention (IR), and skipping exon (SE). **h** Kyoto Encyclopedia of Genes and Genomes (KEGG) pathway enrichment analysis of differential SE and IR expressed genes between ZH11 and SYF2-RNAi9 transcriptome. Significant enrichment was detected with *P* value < 0.05

Considering OsSYF2 is a putative subunit of the spliceosome and impacts grain size, the transcription of *OsSYF2* was detected in different developmental stages of the panicle. Our qRT-PCR results showed that *OsSYF2* had the highest transcription levels in 6-cm panicles (Fig. 2d). Then, 6-cm panicles of ZH11 and Ri9 were harvested and used for RNA-seq analysis. There were 32,502 and 32,228 transcripts detected in the transcriptome of ZH11 and Ri9 panicles*,* respectively (Fig. 5a). Two thousand seven hundred sixty genes were differentially-expressed between Ri9 and ZH11 as determined by a 2-fold cutoff (false discovery rate < 0.05). Of the 2760 differentially-expressed genes, 59.2% (1633/2760) genes were up-regulated, and 40.8% (1127/2760) were down-regulated in Ri9 related to ZH11 (Fig. 5b). The Kyoto Encyclopedia of Genes and Genomes pathway (KEGG) enrichment analysis revealed that the differentially expressed genes were enriched in the functions of RNA transport, mRNA surveillance, RNA degradation, and sugar metabolism (Fig. 5c).

Starch is the primary component of the rice endosperm and considered to be biosynthesized using ADP-glucose as a substrate (Braun et al. 2014). Sugar metabolism and transport influence starch content and morphology (Bai et al. 2016). The contents of sucrose and maltose were decreased in Ri9 and Ri11 lines compared to ZH11 (Fig. 5d, e). Scanning electron microscopy images showed that the starch granules were smaller and more tightly arranged in the endosperm of Ri9 than ZH11 (Fig. 5f). Thus, our data suggested that *OsSYF2* regulates the expression of genes involved in sugar metabolism and affects the formation of starch granules.

### OsSYF2 is Significant for Alternative Splicing

To understand the effects of *OsSYF2* on RNA splicing, the alternative splicing (AS) events were analyzed from our transcriptomic data. Interestingly, the ratio of skipping exon (SE) is increased by 0.6% and the ratio of intron retention (IR) is decreased by 0.4% in Ri9 compared to ZH11 (Fig. 5g). Furthermore, functional assignment of different SE event genes by KEGG analysis indicated that *OsSYF2* SE events were associated with nucleotide excision repair and non-homologous end-joining (Fig. 5h). Meanwhile, the genes with differential IR events were involved in the spliceosome assembly, carbon metabolism, and the pentose phosphate pathway (Fig. 5h). These results indicated that *OsSYF2* plays an important role in alternative splicing.

## Discussion

Grain shape is a complex trait controlled by many factors that include transcription factors, hormones, miRNAs, and cyclophilins (Duan et al. 2015; Ishimaru et al. 2013; Park et al. 2017; Si et al. 2016). Cyclophilins participate in the photosynthetic process, plant growth and development, and stress responses (Cheong et al. 2017; Jing et al. 2015; Park et al. 2013). Here we revealed that the cyclophilin OsCYP20–2 directly interacts with OsSYF2, a subunit of the spliceosome, to regulate grain length in rice. Both the mutation of *OsCYP20–2* and knockdown of *OsSYF2* resulted in short grains. Scanning electron microscopy demonstrated that both genes positively regulate cell length in the lemma (Figs. 1e and 3e). The changes in starch morphology, sucrose content, and maltose content suggested that sugar metabolism was abnormal in the OsSYF2-RNAi lines (Fig. 5e, f). In humans, eight nuclear-located cyclophilins interact with the spliceosome to regulate transcription and pre-mRNA splicing. Among the eight nuclear cyclophilins, peptidyl prolylisomerase-like 2 (PPIL2), spliceosome-associated protein CWC27 homolog (CWC27), peptidyl prolyl isomerase G (PPIG), and peptidyl prolyl isomerase H (PPIH) had more substantial effects on splicing and spliceosome assembly than peptidyl prolyl isomerase E (PPIE), peptidyl prolylisomerase-like 2 (PPIL2), peptidyl prolyl isomerase-like 2 (PPIL3), and peptidyl prolyl isomerase domain and WD repeat-containing protein 1 (PPWD1) (Rajiv and Davis 2018). PPIL1, a minimal signal isomerase domain cyclophilin, interacted with SYF2 assuming to regulate pre-mRNA splicing (Stegmann et al. 2010). Considering that OsSYF2 is a predicted subunit of the rice spliceosome, we examined the splicing patterns of genes related to sugar metabolism. The intron retention and skipping exon events were changed in the OsSYF2-RNAi line relative to the ZH11 control (Fig. 5g, h). Based on these results, we speculated that the interaction between OsCYP20–2 and OsSYF2 is important for mRNA processing and splicing, thereby regulating grain size by way of cell elongation and sugar metabolism.

*OsCYP20–2* and *OsSYF2* had variable expression in different developmental stages of the panicle (Figs. 1f and 2d). Moreover, *OsCYP20–2* and *OsSYF2* are necessary for panicle length regulation in rice (Figs. 1g and 4a). Our transcriptome data showed that *OsSYF2* affects the expression of genes related to RNA surveillance, RNA degradation, RNA transport, and sugar metabolism (Fig. 5c). Comparing the AS events in ZH11 and Ri9, we found that *OsSYF2* majorly impacts the frequency of skipping exon (SE) and intron retention (IR) events (Fig. 5g). The genes with differential SE and IR were enriched for functions in carbon metabolism, spliceosome assembly, and nucleotide excision repair (Fig. 5). Among the differential IR events between ZH11 and Ri9, *LOC_Os03g15050* encodes a phosphoenol/pyruvate carboxykinase (PEPCK), which facilitates the conversion of lipids to sugars (Penfield et al. 2004; Malone et al. 2007). *LOC_Os03g15050* encodes a transaldolase, which plays an important role in glycolysis and the pentose phosphate pathway (PPP) (Caillau and Paul Quick 2005). In addition, *LOC_Os02g44550* (an NADP-dependent malic enzyme) and *LOC_Os07g42440* (a glycolate oxidase) have important functions in photosynthesis (Cui et al. 2016; Tao et al. 2016). It was recently found that splicing factors participated in flower and embryo development in *Arabidopsis* (Xiong et al. 2019a, b), and seed development in maize (Fouquet et al. 2011). Taken together, *OsSYF2* may regulate the frequency of IR and ES events participating in panicle development.

## Conclusions

Overall, we speculated that OsCYP20–2 interacted with OsSYF2 to regulate gene expression and the frequency of AS events to affect grain shape and panicle length. However, how *OsSYF2* affects AS events is still unknown. This study provides further insight into the genetic mechanisms that impact grain yield in rice and provides new targets for molecule breeding to improve rice yield.

## Methods

### Plant Materials and Growth Conditions

The line *oscyp20–2 t1* is a T-DNA insertion mutant in the Hwayoung background (a *japonica* variety of *Oryza sativa*). Two independent RNAi lines (Ri9 and Ri11) and three overexpression lines (OE3, OE6, and OE9) of *OsSYF2* were generated in the Zhonghua 11 (ZH11, a *japonica* variety of *Oryza sativa*) background, separately. The 410-bp fragment from 398 to 807 in the coding sequence of *OsSYF2* and the full-length *OsSYF2* coding sequence were inserted into pTCK303 and pUN1301, respectively, to generate transgenic materials. The 2018 bp sequence upstream of the *OsCYP20–2* coding sequence and the 2067 bp sequence upstream of the *OsSYF2* coding sequence fragment were inserted into pCAMBIA1391z vectors to generate GUS constructs. The GUS constructs were transformed into ZH11 to generate transgenic plants. The young panicles from P2 to P6 stage (Jain et al. 2007) were selected for GUS staining.

Rice plants were cultured in the field in the summer in Beijing (40°06′N), China, under normal conditions, including irrigation and fertilization (Che et al. 2015). Within a row, the distance between the plants was 15 cm, which the distance was 20 cm between the rows. After all measured materials fertilization on October, statistical analysis was performed on agriculture traits of transgenic plants.

### RNA Isolation and qRT-PCR

For identifying the transgenic plants, three-leaf stage seedlings of ZH11 and *OsSYF*2 transgenic lines were selected for RNA isolation via an RNA extraction kit (Qiagen, Germany). Various tissues, including root, young stem, young leaf, mature leaf, flower, panicles of 3–4 cm, 4–5 cm, 6 cm, 8 cm, 10 cm, and 12 cm, were harvested until RNA isolation. Subsequently, 2 μg RNA was used to produce cDNA with SuperScript reverse transcriptase (Invitrogen) with random primers. For qRT-PCR, the *UBIQUITIN* gene was used as an internal control. Each biological sample consisted of three technical replicates. The primers are listed in Table S1.

### Subcellular Localization

To study the subcellular localization of OsSYF2, GFP was fused to the C terminus of OsSYF2 in the pBI221 vector. The plasmids were co-transformed into rice protoplasts, which were released from 10-day-old rice seedlings, with the nuclear marker construct *AtH2B-mCherry* via the polyethylene glycol (PEG 4000) method (Bart et al. 2006). After culturing overnight, the transformed protoplasts were observed with a Leica TCS SP5 fluorescence microscope.

### Yeast Two-Hybrid Assays

The full-length of *OsCYP20–2* and *OsSYF2* ORFs were separately cloned into pGBKT7 and pGADT7, respectively. Then, the constructs were transformed into the yeast strain AH109 and cultured on SD/−Trp-Leu medium. After 3 days, positive clones were selected to culture on SD/−Trp-Leu-His-Ade medium containing 100 μg/mL X-α-gal.

### Scanning Electron Microscopy

Fresh panicles were fixed in FAA at 4 °C overnight and dehydrated in an ethanol series ranging from 75% ~ 100% by every for 30 min at each step. Then, the samples were critical point dried by CO_2_ and imaged on a scanning electron microscope (S-4800 FESEM, Janpan).

### GST-Pull Down

For GST pull-down assays, 50 μL of glutathione sepharose beads (GE Health, Glutathione Sepharose 4B) was washed with 1 × PBS three times. Fifty micrograms of purified GST-CYP20–2 or GST protein were incubated with pre-washing beads overnight at 4 °C. Thirty micrograms of purified pCold-His-SYF2 protein was dissolved in reaction buffer (20 mM Tris, 150 mM NaCl, 2 mM DTT, 5% glycerol, and PMSF) and mixed with Sepharose beads conjugated to GST or GST-CYP20–2 for 2 h at 4 °C. After washing three times, the target protein was centrifuged and analyzed by 10% SDS-PAGE and western blotting with anti-GST or anti-His antibodies.

### Determination of Glucose, Sucrose, and Maltose Content

The mature seeds of ZH11 and OsSYF2-RNAi were dried and peeled. Seeds were ground into a powder and 2 mL ddH_2_O was added to 0.2 g sample, then subjected to ultrasonic extraction for 60 min. Samples were centrifuged at 15,000 rpm for 10 min at room temperature. The supernatant was filtered through the water system and the samples were measured via HPLC (Agilent 1260 Infinity). The velocity of flow was 1 mL/min in Agilent Zorbax NH2 (250 mm*4.6 mm, 5 μm) at room temperature.

### RNA-Seq and Data Analysis

RNA-seq libraries were constructed according to the manufacturer′s protocol. An Illumina HiSeq 2000 platform was selected for RNA-seq by the Beijing Genomics Institute. After discarding the low-quality reads, B_OWTIE_ was used to rebuild the genome, which used the MSU Rice Genome Annotation Project Release 7 (http://rice.plantbiology.msu.edu/) as the reference genome.

MeV v4.9 (Saeed et al. 2003) was used to generate heat maps showing gene expression levels. Kyoto Encyclopedia of Genes and Genomes (KEGG) pathways analysis was performed using KOBAS v.2.0 (Xie et al. 2011). Significant enrichment was detected with a *P* value < 0.05.

## Supplementary information

**Additional file 1: Supplemental Table S1.** Primers used in this study.

## Data Availability

The datasets in this study are available from the corresponding author on reasonable request.
